# Fungal Diversity and Community Composition across Ecosystems

**DOI:** 10.3390/jof9050510

**Published:** 2023-04-25

**Authors:** Pavla Debeljak, Federico Baltar

**Affiliations:** 1 Fungal & Biogeochemical Oceanography, Department of Functional and Evolutionary Ecology, University of Vienna, 1030 Vienna, Austria; 2 SupBiotech, 94800 Villejuif, France

**Keywords:** 18S rRNA, fungal diversity, amplicon sequencing variants (ASVs), marine fungi, ecosystems

## Abstract

Fungi have shaped the biosphere since the development of life on Earth. Despite fungi being present in all environments, most of the available fungal research has focused on soils. As a result, the role and composition of fungal communities in aquatic (marine and freshwater) environments remain largely unexplored. The use of different primers to characterise fungal communities has additionally complicated intercomparisons among studies. Consequently, we lack a basic global assessment of fungal diversity across major ecosystems. Here, we took advantage of a recently published 18S rRNA dataset comprising samples from major ecosystems (terrestrial, freshwater, and marine) to attempt a global assessment of fungal diversity and community composition. We found the highest fungal diversities for terrestrial > freshwater > marine environments, and pronounced gradients of fungal diversity along temperature, salinity, and latitude in all ecosystems. We also identified the most abundant taxa in each of these ecosystems, mostly dominated by Ascomycota and Basidiomycota, except in freshwater rivers where Chytridiomycota dominated. Collectively, our analysis provides a global analysis of fungal diversity across all major environmental ecosystems, highlighting the most distinct order and ASVs (amplicon sequencing variants) by ecosystem, and thus filling a critical gap in the study of the Earth’s mycobiome.

## 1. Introduction

Fungi represent a large part of the global genetic diversity, and their evolution and diversification had major implications for the development of life on Earth [1,2,3,4]. The total diversity of fungi has currently been estimated to be 2.2–3.8 million species [1,5,6]. These so-called mycobiomes are generally predominated by three phyla: the Ascomycota, Basidiomycota, and Chytridiomycota in marine ecosystems [7], while terrestrial ecosystems are generally dominated by Ascomycota and Basidiomycota [8].

In terrestrial and freshwater ecosystems, fungi are one of the key organismal groups necessary for the cycling of plant detritus, contributing to key elemental cycles by releasing CO_2_ into the atmosphere and inorganic nitrogen and phosphorous into the soil [9]. Previous analysis of soil samples using 454 sequencing technology on the internal transcribed spacer (ITS) region identified distance from the Earth’s equator and mean annual precipitation as factors with the strongest effects on the richness of soil fungi [8]. Less is known about the ecology of aquatic fungi (freshwater and marine), although recent evidence indicates they also play a critical role in those environments [9]. Although most of the studies on marine fungal ecology have been associated with debris such as driftwood or seafloor sediments [9], recent evidence suggests that fungi are also present in the oceanic water column exhibiting biomass levels as high as prokaryotes on particles [9,10,11,12,13]. A recent attempt focusing on samples from the marine environment using 18S rRNA found a diverse community composition that was influenced by salinity [7].

Yet, most of the research on fungal diversity has focused on genetic and molecular studies from soil environments, and the question of how fungal biodiversity is partitioned across different ecosystems as well as temporal and spatial scales remains unresolved. Moreover, different primers are frequently used to characterise fungal communities, complicating intercomparisons among studies. Therefore, a global integrative analysis including terrestrial, freshwater, and marine ecosystems is urgently lacking. This gap becomes even more relevant in light of recent findings highlighting the critical importance of fungi not only in soils but also in aquatic ecosystems [9,10,14,15,16,17]. To reduce this gap, here we used a recently published compilation of metabarcoding studies allowing for large-scale comparisons of eukaryotic microorganisms across ecosystems, which focuses on the V4 region of the 18S rRNA as ASVs [18]. We hypothesised to find different fungal diversities and community assemblages across systems, with higher diversities in soils than in aquatic environments.

## 2. Materials and Methods

Data retrieval. Data were retrieved from the metaPR2 web browser accessed in February 2022 (https://shiny.metapr2.org/metapr2/, accessed on 4 March 2022) [18]. Users need to ‘enter’ the website portal in order to access the database. The following samples were collected: V4 gene regions, DNA and RNA, all available ecosystems, all available substrates, and all available size fractions and depth levels for the Opisthokonta, including the Kingdom of Fungi. The data were downloaded as an RDS element that was directly imported into R using the command ‘readRDS(“metapr2_phyloseq_ALLFungi_2022-03-01.rds”, refhook = NULL)’. All following analyses were performed in the R version 4.1.3 (2022-03-10)—“One Push-Up”.

Statistical Analysis. The RDS format was a phyloseq object which is an R package [19] to import, store, analyse, and graphically display complex phylogenetic sequencing data that has already been clustered into Amplicon sequencing variants (ASVs) or more appropriately denoised, and it is most useful when there is also associated sample data, phylogeny, and/or taxonomic assignment of each taxon. phyloseq leverages and builds upon many of the tools available in R for ecology and phylogenetic analysis (vegan13, ade414, ape15), while also using advanced/flexible graphic systems (ggplot216) [20] to easily produce publication-quality graphics of complex phylogenetic data. The phyloseq package uses a specialised system of S4 data classes to store all related phylogenetic sequencing data as a single, self-consistent, self-describing experiment-level object, making it easier to share data and reproduce analyses. All multivariate statistical analyses were performed in the vegan package in R [21]. Diveristy plots were plotted using the plot_bar() function in phyloseq. The metadata (all environmental parameters) to the dataset were included in the downloaded RDS file.

DESeq2. Statistical significance of differences in ASV relative abundances between treatments was determined using DESeq2 (version 1.10.1) [22]. The workflow described as part of the phyloseq addition with DeSeq2 was used (https://joey711.github.io/phyloseq-extensions/DESeq2.html, accessed on 4 March 2022) and modified following the methodology used by Pelikan et al. [23]. Only ASVs that had ≥5 reads and that were present in ≥5 of the samples per ecosystem were kept. The ASV table from phyloseq was used as raw count data for DESeq2; however, all 0 values had to be transformed to 1. Results were extracted with the command: results(cooksCutoff  =  FALSE, contrast = c(“ecosystem”,”terrestrial”,”oceanic”)) and were considered statistically significant if the false-discovery-rate (FDR)-adjusted *p* value was below 0.05.

Data visualisation and availability. All Figures were produced in R using ggplot [20] and exported as svg formats, and merged in the open-source program Inkscape (https://inkscape.org/, accessed on 4 March 2022). All analysis files, as well as R markdown files, can be found on the publicly accessible GitHub page https://github.com/PavlaDe/ASV_FungiFun (accessed on 4 March 2022).

## 3. Results and Discussion

### 3.1. Global Comparison of Fungal Diversity by Ecosystem

**Figure 1:**
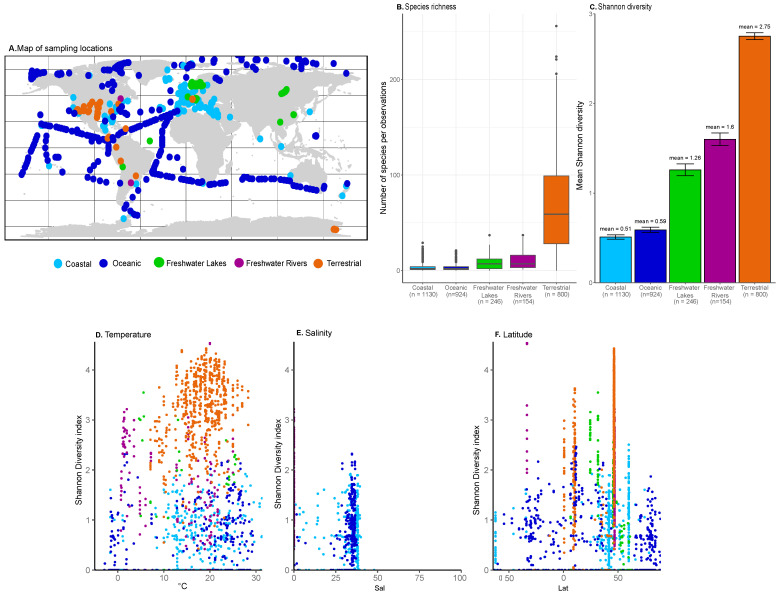
(**A**) Sampling locations from 41 campaigns and expeditions with presence of fungal ASVs. (**B**) Species richness by ecosystem; (**C**) Shannon diversity by ecosystem; (**D**) Shannon diversity by temperature (°C); (**E**) Shannon diversity by salinity; (**F**) Shannon diversity by latitude.

The fungi dataset comprised 2332 samples compiled from 41 independent terrestrial, coastal, and oceanic sampling campaigns (Figure 1A). This dataset is extensive, although there is a high difference in sampling effort that is to be expected due to the heterogeneity of the datasets. The coastal dataset was the largest, with 1130 samples, followed by the oceanic and terrestrial (n = 924 and n = 800, respectively), and the smaller freshwater datasets (freshwater rivers, n = 154; and freshwater lakes, n = 246). The number of species per observation was clearly highest for terrestrial ecosystems, with a mean value of 57 fungal taxa by sample (Figure 1B). This was also represented by the highest Shannon diversity, which was significantly different (*p* < 0.001, ANOVA) between the five ecosystems, ranging from 2.75 in terrestrial to 1.6–1.26 in freshwater ecosystems, and down to 0.59–0.51 in marine systems (Figure 1C). Our findings confirm the assumption that terrestrial fungal diversity is highest and decreases towards aquatic ecosystems, although this observation might be biased by the lack of large-scale available data for fungi from aquatic ecosystems [24]. In the marine environment, the highest fungal diversities are often found in surface waters [25] and close to the coast [26,27,28], with the lowest diversity at oceanic sites [28], although not always; e.g., no significant differences were found for fungal diversity in a recent study along a transect covering estuary to oligotrophic waters in the Sargasso Sea [29].

We retrieved environmental parameters from the published database and focused our analysis on those common to all ecosystems. To understand the influence of environmental factors on fungal diversity, we tested salinity, temperature, and latitude as environmental indicators for the different ecosystems (Figure 1D–F). We focused on the specific V4 region of the 18S rRNA and calculated alpha diversity, inverse Simpson, and the Chao estimator. These further confirmed the observed diversity observations (Supplementary Figure S1). Maximum alpha diversities were found in terrestrial systems, increasing from 8.2 to 19.7 °C and then decreasing towards 30 °C (Figure 1D, Supplementary Figure S1A). In contrast, the highest diversities in rivers were found between 1.3 and 7 °C. Marine fungi communities increased in diversity with increasing salinity up to 37, while the highest diversities for rivers were found at 0 salinity (Figure 1E, Supplementary Figure S1B). The highest terrestrial fungal diversity was observed around 50 °N, contrasting previously observed highest diversity at the equator [8]. Interestingly, marine fungal diversity generally decreased towards high latitudes (Figure 1F, Supplementary Figure S1C), which is in line with observed decreases for fungal communities [13] as well as marine bacteria and phytoplankton diversity with increasing latitude [30,31,32].

### 3.2. Fungal Communities by Ecosystem

**Figure 2:**
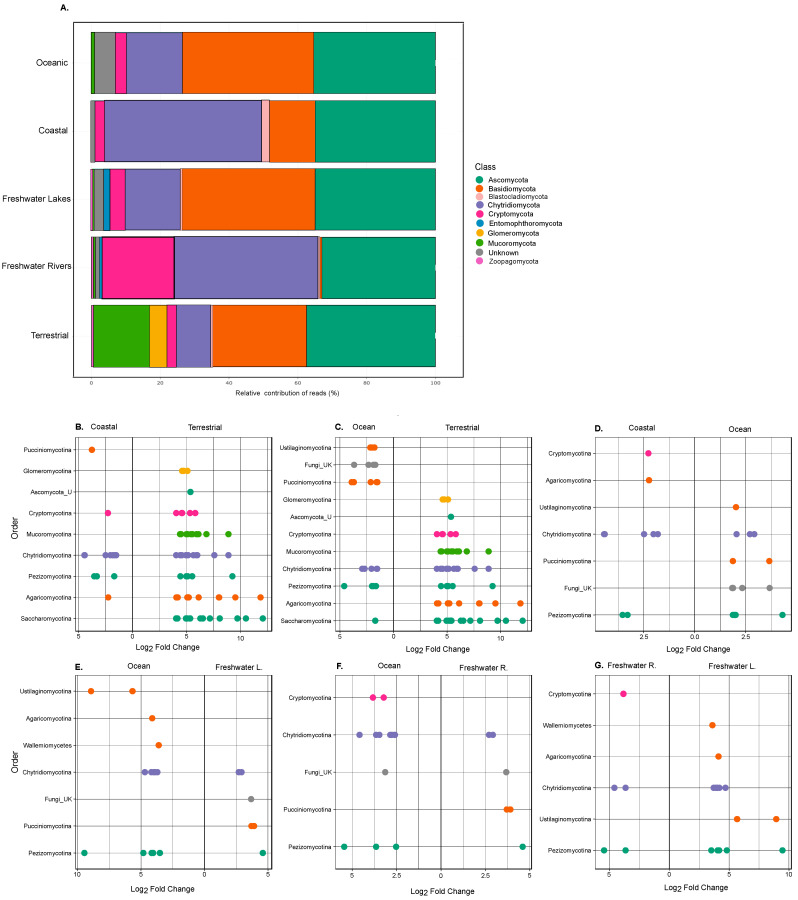
(**A**) Relative contribution of reads by ecosystem according to fungal class annotation. (**B**–**G**) Deseq2 Analysis was performed to highlight the differences in fungal order by ecosystem. For each comparison, the greatest log2 fold changes are depicted. Note the difference in Log2 Fold change per comparison.

We further identified the main contributors to fungal diversity across ecosystems. In all ecosystems, the dominating fungal phyla were Ascomycota and Basidiomycota, followed by Chytridiomycota, with the highest contribution of Cryptomycota found in freshwater rivers (16.7%) (Figure 2A, Supplementary Figures S2 and S3). In terrestrial communities, Mucoromycota (16.6%) and Glomeromycota (4.8%) also contributed substantially. There was also a relevant contribution of fungi that are not classified (termed ‘Unkn’) in all ecosystems, particularly in the freshwater river (1.4%), lake (3.2%), coastal (0.9%), and oceanic ecosystems (5.8%). Although it is difficult to compare our relative abundances to previous work due to the lack of comparative analyses across multiple ecosystems, the main subphyla and class contributions obtained are in consensus to specific soil [8], marine [7,13], or freshwater datasets [33]. Analysis of Variance Using Distance Matrices (‘Adonis’ function in R) revealed that the communities were significantly different in their taxa composition between ecosystems. This was further confirmed by principal component analysis (PCA) (Supplementary Figure S4).

**Figure 3:**
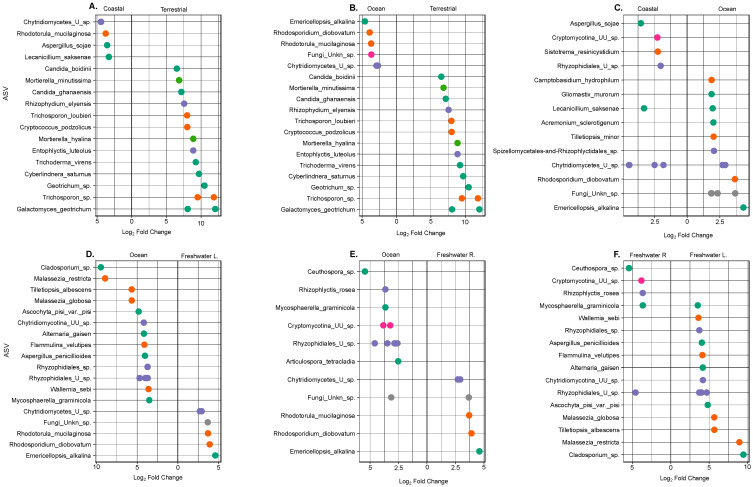
(**A**–**F**) Deseq2 Analysis was performed to highlight the differences in ASVs by ecosystem. For each comparison, the greatest log2 fold changes are depicted. Note the difference in Log2 Fold change per comparison.

In order to identify specific differences in community composition between ecosystems, we performed differential expression analysis, modified for ASV count data, and focused on the taxonomic level of order (Figure 2). We further repeated the analysis with the genus-specific ASVs (Figure 3), although we acknowledge that the genus-level ASV-specific differences need to be taken with care due to the difficulty in differentiating genera from the sequencing of the 18S rRNA region [34].

The subphyla from coastal datasets with the highest differences (represented by greatest log_2_ fold changes) to terrestrial communities were Pucciniomycotina, Cryptomycotina, and Agaricomycotina (Figure 2B). For ocean datasets the subphyla Ustilaginomycotina, Pucciniomycotina, and not classified fungi had highest log2 fold changes to terrestrial communities (Figure 2C). All of these have been previously found in coastal and oceanic environments [35]. Members of the Ustilaginomycotina were described as marine smut fungi and have been isolated from anoxic zones of the Arabian Sea [36,37]. Pezizomycotina have shown to be dominant fungal symbionts of sponges together with Agaricomycotina in marine environments [38,39]. The coastal communities differentiated from the oceanic ones mostly by the subphyla Cryptomycotina and Agaricomycotina compared to Pucciniomycotina and Ustilaginomycotina (Figure 2D). Agaricomycotina have additionally been found to be associated with seaweeds in coastal waters [40]. Oceanic fungal communities exhibited the highest differences from freshwater lake communities in Ustilaginomycotina, Agaricomycotina, and the class Wallemiomycetes (Figure 2E), and from freshwater river communities in Cryptomycotina (Figure 2F). While the differences in freshwater lake communities compared to freshwater rivers were strongest in Wallemiomycetes, Agrarimycotina, and Ustilaginomycotina (Figure 2G).

### 3.3. ASV-Specific Differences by Ecosystem

The marine communities differentiated from the terrestrial ones by several specific ASVs, mostly *Rhodotorula mucilaginosa* (Figure 3A,B) and an undefined Chytridiomycota. The highest difference between oceanic datasets to coastal datasets was observed for *Emericellopsis alkalina* and *Rhodosporidium diobovatum* (Figure 3C). *Rhodotorula* species are predominant basidiomycete yeasts in the marine environment [14]. *Rhodotorula mucilaginosa* has been isolated from a sediment core at 3600 m depth and cultivated at atmospheric pressure. It is a fungal species of interest due to its ability to produce valuable natural products, such as lipids and carotenoids, with potential applications as surfactants, food additives, and pharmaceuticals [41]. Chytridiomycota have been defined as one of the dominant groups of parasites in aquatic ecosystems. The free-living zoosporic stage of Chytridiomycota actively searches for and infects host cells, extracting nutrients and developing into mature sporangia that release new zoospores [42]. These zoospores are a good food source for zooplankton in terms of size and shape and led to the development of the “mycoloop” concept [28]. When large inedible phytoplankton species are infected by chytridiomycota, nutrients within host cells are transferred to zooplankton via the zoospores of parasitic chytridiomycota. The “mycoloop” may play an important role in shaping aquatic ecosystems by altering sinking fluxes or determining system stability [43]. The identification of zoospores remains challenging, and the presence of these taxa in relation to the terrestrial datasets highlights their importance in marine ecosystems.

The highest difference between oceanic datasets to coastal datasets was observed for *Emericellopsis alkalina* and *Rhodosporidium diobovatum* (Figure 3C). *Emericellopsis alkalina* has foremost been described in alkali-tolerant soils with a growth optimum at pH above 9. The molecular adaptations to these alkaline conditions are of high interest for biotechnology, and the similarity of other *Emericellopsis* to the marine taxa has raised the hypothesis of its marine origin [44]. Its adaptation to alkaline environments could potentially explain its presence in the oceanic dataset that experiences changes in pH with depth. *Rhodosporidium diobovatum* was slated from marine and estuarine waters as well as deep-sea sediments and is defined as a relationship between the yeast form genus *Rhodotorula* and heterobasidiomycetous fungi [45,46].

Terrestrial differences from marine systems (oceanic and coastal) were fairly similar, with the most distinct ASVs being *Geotrichum* sp., *Trichosporon* sp., and *Galactomyces geotrichum* (Figure 3A,B). All three of these have been observed in soil samples from different parts of the world and are defined as soil fungi [8,24]. The fungal taxa explaining most of the variation in the terrestrial community was *Mortierella hyalina* belonging to the Mucoromycotina subphylum, which was significantly different from the marine samples (Figure 3A,B). *Mortierella hyalina* was also the driving ASV for terrestrial communities in the PCA, showing that the communities were significantly different in their taxa composition between ecosystems (Supplementary Figure S4). *Mortierella hyalina* is a known beneficial root-colonising fungus [47], and the morphological diversity of the *Mortierella* genus remains underestimated [48]. Given the plant association and filamentous nature, this species has been observed in the highest abundance in most soils from different ecosystems [8].

In freshwater lakes, the differences from oceanic communities were mostly due to *Cladosporium* sp. and *Malassezia restricta* (Figure 3D). Rivers, on the other hand, exhibited differences from oceanic ecosystems and freshwater lakes in two members of the Ascomycota, namely *Ceuthospora* sp., *Mycosphaerella graminicola,* as well as and the Chytridomycota member, *Rhizophlyctis rosea* (Figure 3E,F). These results are consistent with ecosystem-specific studies where each of those fungal taxa has been shown to be present and/or dominate in their corresponding ecosystem [44,46]. Many aquatic habitats construe a submersion gradient from land to water along a floodplain, and since the precise positions of such gradients may fluctuate seasonally and/or episodically with weather events, it is difficult to establish precise aquatic boundaries [49]. Additionally, the degree of submergence of the substrates of freshwater ascomycetes, for example, can vary with fluctuations in water level. Whether these taxa are truly freshwater species and not of terrestrial origin is difficult to assess [49]. This is also reflected in the number of shared ASVs between freshwater ecosystems to terrestrial ecosystems (Figure 3F).

Taken together, our results provide the exploration of fungal diversity on the order and ASV level across major ecosystems. We found that fungal diversities decrease from terrestrial > freshwater > marine environments. We also revealed the most distinct taxa by ecosystem, and concluding that the communities are shaped by environmental parameters resulting in ASV-specific communities. We acknowledge the difficulties associated with potential database heterogeneity; nonetheless, this study provides a base for ecosystem intercomparisons of fungal diversity. The increase in deposited sequence data will fuel future studies to describe a more complete picture of global fungal diversity.

## Data Availability

The input files for analysis, as well as the R Markdown file, are publicly available on GitHub page as well as fasta files for ASVs (https://github.com/PavlaDe/ASV_FungiFun, accessed on 4 March 2022). The download process, as well as the subsequent steps, are documented in the R Markdown files and are reproducible using the input files.

## Supplementary Materials

The following supporting information can be downloaded at https://www.mdpi.com/article/10.3390/jof9050510/s1. Figure S1: Alpha diversity metrics (Shannon and Inverse Simpson) as well as Chao estimators; Figure S2: Prevalence (Abundance of ASVs by observed samples) of ASVs by ecosystem and fungal class; Figure S3: Boxplots of relative abundances of fungal family by ecosystem; Figure S4: Principal Component Analysis on bray-curtis similarity matrices from different ecosystems.

## References

[1] Hawksworth D.L. (1991). The Fungal Dimension of Biodiversity: Magnitude, Significance, and Conservation. Mycol. Res..

[2] Hibbett D.S., Binder M., Bischoff J.F., Blackwell M., Cannon P.F., Eriksson O.E., Huhndorf S., James T., Kirk P.M., Lücking R. (2007). A Higher-Level Phylogenetic Classification of the Fungi. Mycol. Res..

[3] Mueller G.M., Schmit J.P., Leacock P.R., Buyck B., Cifuentes J., Desjardin D.E., Halling R.E., Hjortstam K., Iturriaga T., Larsson K.-H. (2007). Global Diversity and Distribution of Macrofungi. Biodivers. Conserv..

[4] O’Brien H.E., Parrent J.L., Jackson J.A., Moncalvo J.-M., Vilgalys R. (2005). Fungal Community Analysis by Large-Scale Sequencing of Environmental Samples. Appl. Environ. Microbiol..

[5] Hawksworth D.L. (2001). The Magnitude of Fungal Diversity: The 1.5 Million Species Estimate Revisited. Mycol. Res..

[6] Hawksworth D.L., Lücking R. (2017). Fungal Diversity Revisited: 2.2 to 3.8 Million Species. Microbiol. Spectr..

[7] Hassett B.T., Vonnahme T.R., Peng X., Jones E.B.G., Heuzé C. (2020). Global Diversity and Geography of Planktonic Marine Fungi. Bot. Mar..

[8] Tedersoo L., Bahram M., Põlme S., Kõljalg U., Yorou N.S., Wijesundera R., Ruiz L.V., Vasco-Palacios A.M., Thu P.Q., Suija A. (2014). Global Diversity and Geography of Soil Fungi. Science.

[9] Grossart H.-P., Van den Wyngaert S., Kagami M., Wurzbacher C., Cunliffe M., Rojas-Jimenez K. (2019). Fungi in Aquatic Ecosystems. Nat. Rev. Microbiol..

[10] Orsi W.D., Vuillemin A., Coskun Ö.K., Rodriguez P., Oertel Y., Niggemann J., Mohrholz V., Gomez-Saez G.V. (2022). Carbon Assimilating Fungi from Surface Ocean to Subseafloor Revealed by Coupled Phylogenetic and Stable Isotope Analysis. ISME J..

[11] Edgcomb V.P., Beaudoin D., Gast R., Biddle J.F., Teske A. (2011). Marine Subsurface Eukaryotes: The Fungal Majority. Environ. Microbiol..

[12] Bochdansky A.B., Clouse M.A., Herndl G.J. (2017). Eukaryotic Microbes, Principally Fungi and Labyrinthulomycetes, Dominate Biomass on Bathypelagic Marine Snow. ISME J..

[13] Morales S.E., Biswas A., Herndl G.J., Baltar F. (2019). Global Structuring of Phylogenetic and Functional Diversity of Pelagic Fungi by Depth and Temperature. Front. Mar. Sci..

[14] Richards T.A., Jones M.D.M., Leonard G., Bass D. (2012). Marine Fungi: Their Ecology and Molecular Diversity. Annu. Rev. Mar. Sci..

[15] Baltar F., Zhao Z., Herndl G.J. (2021). Potential and Expression of Carbohydrate Utilization by Marine Fungi in the Global Ocean. Microbiome.

[16] Breyer E., Zhao Z., Herndl G.J., Baltar F. (2022). Global Contribution of Pelagic Fungi to Protein Degradation in the Ocean. Microbiome.

[17] Chrismas N., Cunliffe M. (2020). Depth-Dependent Mycoplankton Glycoside Hydrolase Gene Activity in the Open Ocean—Evidence from the Tara Oceans Eukaryote Metatranscriptomes. ISME J..

[18] Vaulot D., Sim C.W.H., Ong D., Teo B., Biwer C., Jamy M., Lopes Dos Santos A. (2022). MetaPR^2^: A Database of Eukaryotic 18S RRNA Metabarcodes with an Emphasis on Protists. Mol. Ecol. Resour..

[19] McMurdie P.J., Holmes S. (2013). Phyloseq: An R Package for Reproducible Interactive Analysis and Graphics of Microbiome Census Data. PLoS ONE.

[20] Wickham H. (2009). Ggplot2: Elegant Graphics for Data Analysis; Use R!.

[21] Dixon P. (2003). VEGAN, A Package of R Functions for Community Ecology. J. Veg. Sci..

[22] Love M.I., Huber W., Anders S. (2014). Moderated Estimation of Fold Change and Dispersion for RNA-Seq Data with DESeq2. Genome Biol..

[23] Pelikan C., Wasmund K., Glombitza C., Hausmann B., Herbold C.W., Flieder M., Loy A. (2021). Anaerobic Bacterial Degradation of Protein and Lipid Macromolecules in Subarctic Marine Sediment. ISME J..

[24] Peay K.G., Kennedy P.G., Talbot J.M. (2016). Dimensions of Biodiversity in the Earth Mycobiome. Nat. Rev. Microbiol..

[25] Li W., Wang M., Burgaud G., Yu H., Cai L. (2019). Fungal Community Composition and Potential Depth-Related Driving Factors Impacting Distribution Pattern and Trophic Modes from Epi- to Abyssopelagic Zones of the Western Pacific Ocean. Microb. Ecol..

[26] Wang X., Singh P., Gao Z., Zhang X., Johnson Z.I., Wang G. (2014). Distribution and Diversity of Planktonic Fungi in the West Pacific Warm Pool. PLoS ONE.

[27] Wang Y., Sen B., He Y., Xie N., Wang G. (2018). Spatiotemporal Distribution and Assemblages of Planktonic Fungi in the Coastal Waters of the Bohai Sea. Front. Microbiol..

[28] Sen K., Bai M., Sen B., Wang G. (2021). Disentangling the Structure and Function of Mycoplankton Communities in the Context of Marine Environmental Heterogeneity. Sci. Total Environ..

[29] Duan Y., Xie N., Wang Z., Johnson Z.I., Hunt D.E., Wang G. (2021). Patchy Distributions and Distinct Niche Partitioning of Mycoplankton Populations across a Nearshore to Open Ocean Gradient. Microbiol. Spectr..

[30] Fuhrman J.A., Steele J.A., Hewson I., Schwalbach M.S., Brown M.V., Green J.L., Brown J.H. (2008). A Latitudinal Diversity Gradient in Planktonic Marine Bacteria. Proc. Natl. Acad. Sci..

[31] Sul W.J., Oliver T.A., Ducklow H.W., Amaral-Zettler L.A., Sogin M.L. (2013). Marine Bacteria Exhibit a Bipolar Distribution. Proc. Natl. Acad. Sci. USA.

[32] Barton A.D., Dutkiewicz S., Flierl G., Bragg J., Follows M.J. (2010). Patterns of Diversity in Marine Phytoplankton. Science.

[33] El-Elimat T., Raja H.A., Figueroa M., Al Sharie A.H., Bunch R.L., Oberlies N.H. (2021). Freshwater Fungi as a Source of Chemical Diversity: A Review. J. Nat. Prod..

[34] Schoch C.L., Seifert K.A., Huhndorf S., Robert V., Spouge J.L., Levesque C.A., Chen W., Bolchacova E., Fungal Barcoding Consortium, Fungal Barcoding Consortium Author List (2012). Nuclear Ribosomal Internal Transcribed Spacer (ITS) Region as a Universal DNA Barcode Marker for *Fungi*. Proc. Natl. Acad. Sci. USA.

[35] Raghukumar S. (2017). Fungi in Coastal and Oceanic Marine Ecosystems.

[36] Bauer R., Lutz M., Piątek M., Vánky K., Oberwinkler F. (2007). *Flamingomyces* and *Parvulago*, New Genera of Marine Smut Fungi (*Ustilaginomycotina*). Mycol. Res..

[37] Manohar C.S., Boekhout T., Müller W.H., Stoeck T. (2014). Tritirachium Candoliense Sp. Nov., a Novel Basidiomycetous Fungus Isolated from the Anoxic Zone of the Arabian Sea. Fungal Biol..

[38] Jin L., Liu F., Sun W., Zhang F., Karuppiah V., Li Z. (2014). *Pezizomycotina* Dominates the Fungal Communities of South China Sea Sponges *Theonella swinhoei* and *Xestospongia testudinaria*. FEMS Microbiol. Ecol..

[39] Baker P.W., Kennedy J., Dobson A.D.W., Marchesi J.R. (2009). Phylogenetic Diversity and Antimicrobial Activities of Fungi Associated with Haliclona Simulans Isolated from Irish Coastal Waters. Mar. Biotechnol..

[40] Francis M., Webb V., Zuccarello G. (2016). Marine Yeast Biodiversity on Seaweeds in New Zealand Waters. N. Z. J. Bot..

[41] Buedenbender L., Kumar A., Blümel M., Kempken F., Tasdemir D. (2020). Genomics- and Metabolomics-Based Investigation of the Deep-Sea Sediment-Derived Yeast, Rhodotorula Mucilaginosa 50-3-19/20B. Mar. Drugs.

[42] Canter H.M. (1967). Studies on British Chytrids: XXVI. A Critical Examination of Zygorhizidium Melosirae Canter and Z. Planktonicum Canter. J. Linn. Soc. Lond. Bot..

[43] Kagami M., Miki T., Takimoto G. (2014). Mycoloop: Chytrids in Aquatic Food Webs. Front. Microbiol..

[44] Grum-Grzhimaylo A.A., Georgieva M.L., Debets A.J.M., Bilanenko E.N. (2013). Are Alkalitolerant Fungi of the *Emericellopsis* Lineage (*Bionectriaceae*) of Marine Origin?. IMA Fungus.

[45] Newell S.Y., Hunter I.L. (1970). *Rhodosporidium diobovatum* sp. n., the Perfect Form of an Asporogenous Yeast (*Rhodotorula* sp.). J. Bacteriol..

[46] Zeng L.-P., Huang J.-F., Qiu G.-Z., Chu F.-Y., Chen D., Tong J.-B., Luo X.-G. (2009). Isolation and Identification of Rhodosporidium Diobovatum DS-0205 from Deep-Sea Sediment of Eastern Pacific Ocean. J. Cent. South Univ. Technol..

[47] Johnson J.M., Ludwig A., Furch A.C.U., Mithöfer A., Scholz S., Reichelt M., Oelmüller R. (2019). The Beneficial Root-Colonizing Fungus *Mortierella hyalina* Promotes the Aerial Growth of *Arabidopsis* and Activates Calcium-Dependent Responses That Restrict *Alternaria brassicae*-Induced Disease Development in Roots. Mol. Plant-Microbe Interact..

[48] Lee J.-S., Nam B., Lee H.B., Choi Y.-J. (2018). Molecular Phylogeny and Morphology Reveal the Underestimated Diversity of Mortierella (Mortierellales) in Korea. Korean J. Mycol..

[49] Shearer C.A., Raja H.A., Miller A.N., Nelson P., Tanaka K., Hirayama K., Marvanová L., Hyde K.D., Zhang Y. (2009). The Molecular Phylogeny of Freshwater Dothideomycetes. Stud. Mycol..

