# Supplementary material for: Fungal Diversity and Community Composition across Ecosystems
Source: J Fungi (Basel). 2023 Apr 25;9(5):510. doi: 10.3390/jof9050510 (PMC10219167; doi:10.3390/jof9050510)

Supplementary Figure S1. Alpha diversity metrics (Shannon and Inverse Simpson) as well as Chao estimators by **A.** temperature **B.** salinity **C.** latitude

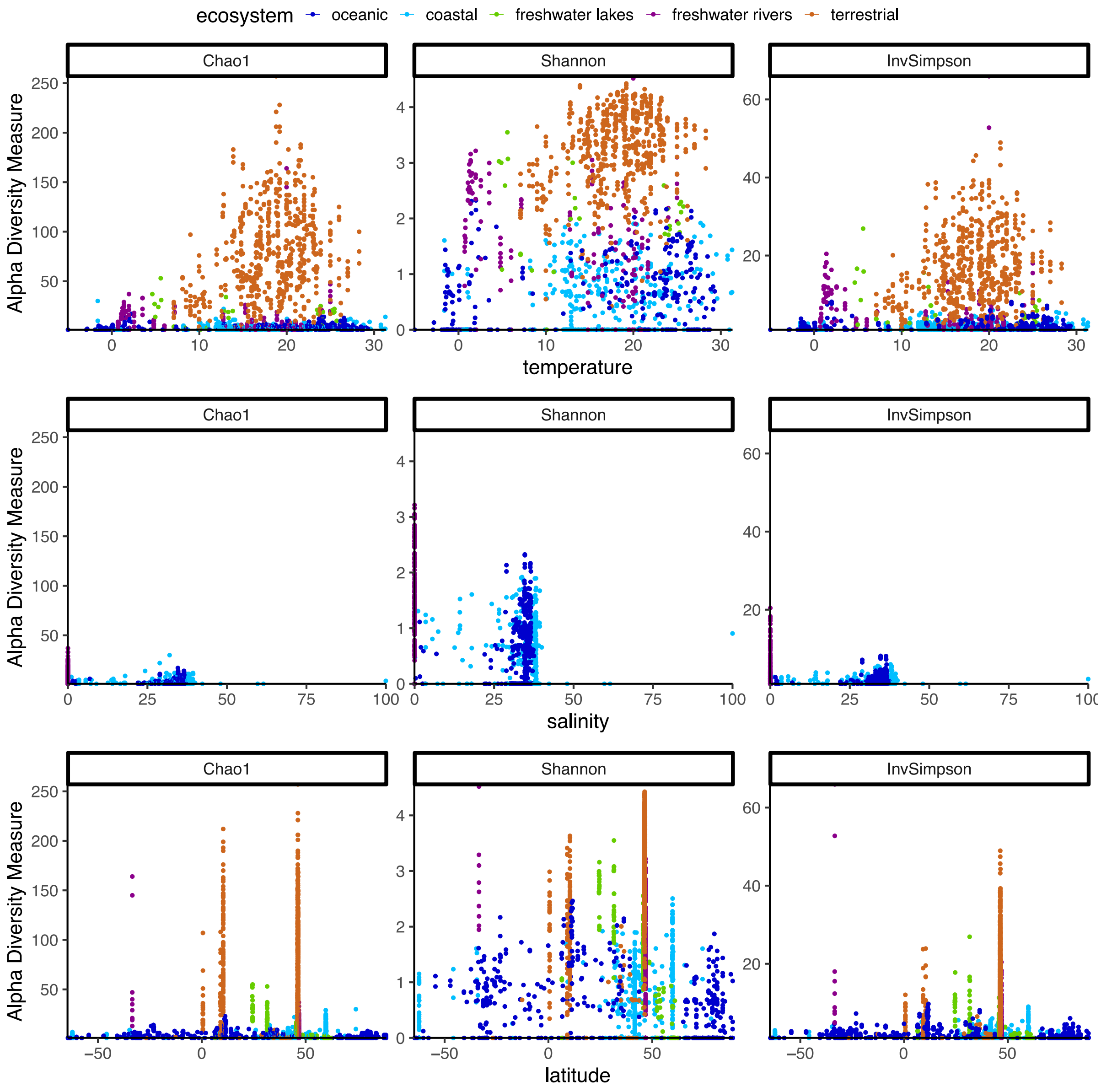

Supplementary Figure S2. Prevalence (Abundance of ASVs by observed samples) of ASVs by ecosystem and fungal class.

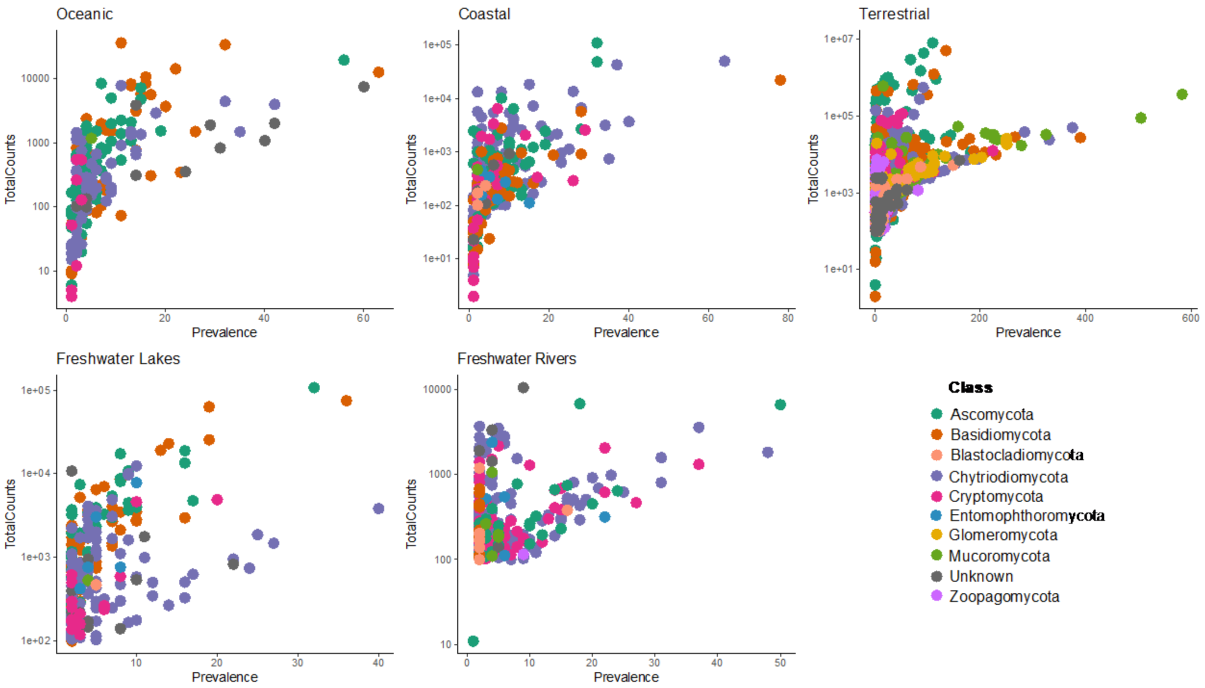

Supplementary Figure S3. A-E. Boxplots of relative abundances of fungal family by ecosystem.

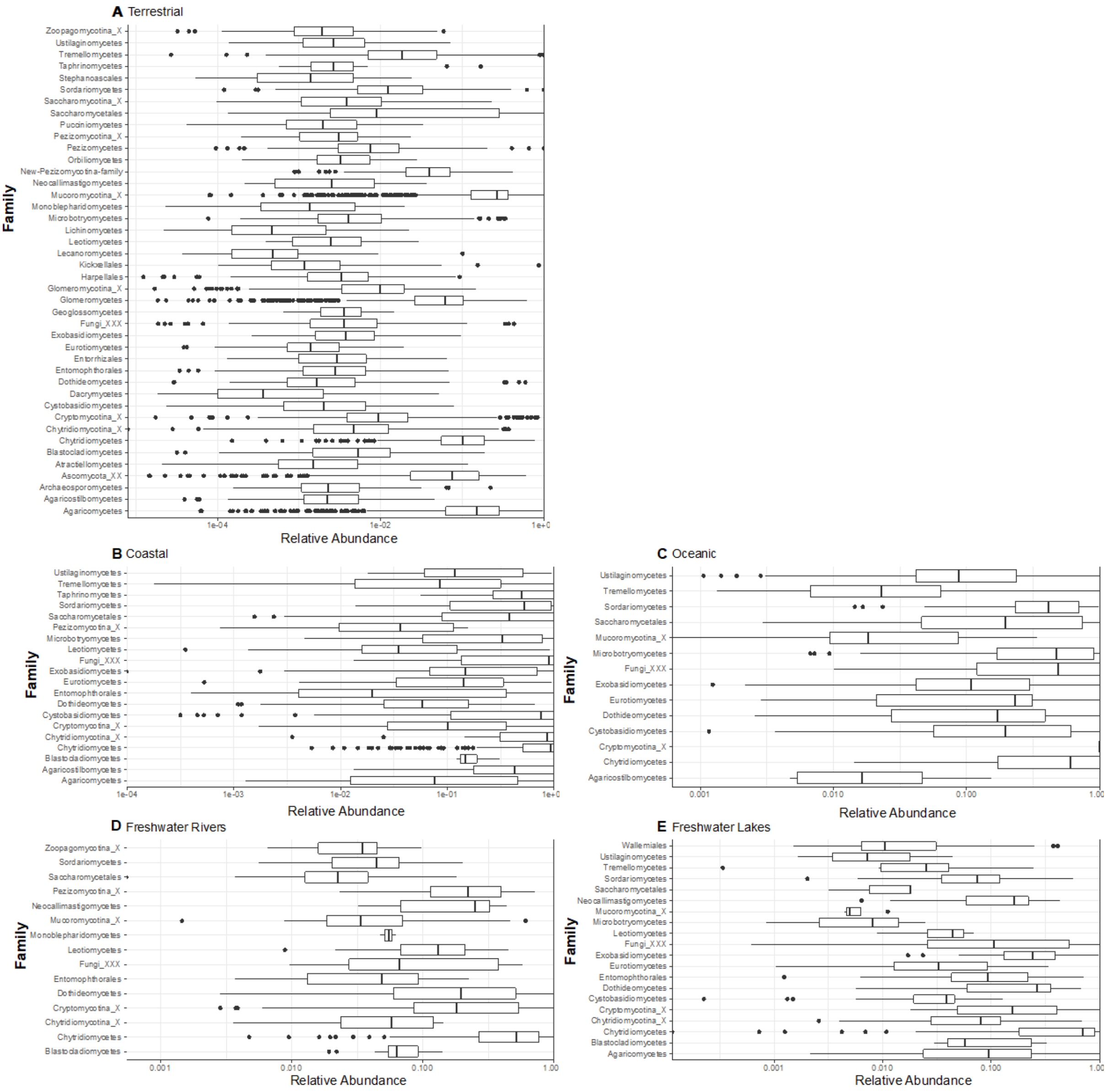

Supplementary Figure S4. Principal Component Analysis on bray-curtis similarity matrices from different ecosystems. Visible ASVs represent '3a575d49' - Mortierella_hyalina, 'fba3dc651' - Rhodotorula_mucilaginosa, '3b78a83cc9' - Chytridiomycetes_X_sp., '544bf132f8' - Chytridiomycetes_X_sp., '308b32461' - Fungi_Unkn_sp.. All other ASVs are provided in separate Excel sheet (asv.xlsx).

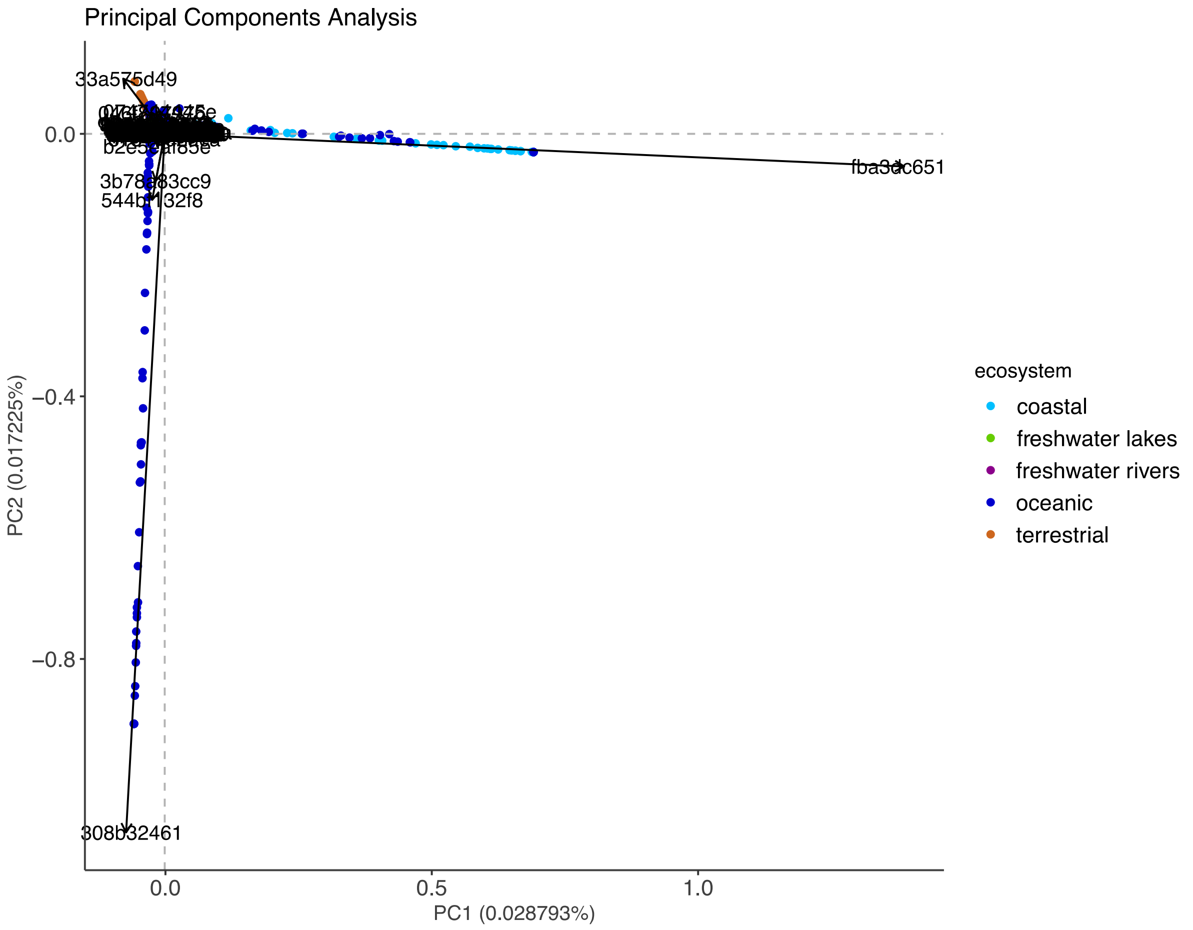

Supplement: Supplementary file 1 [file jof-09-00510-s001.zip › jof-2310852-supplementary.pdf]
